# Depletion of Murine Intestinal Microbiota: Effects on Gut Mucosa and Epithelial Gene Expression

**DOI:** 10.1371/journal.pone.0017996

**Published:** 2011-03-21

**Authors:** Dag Henrik Reikvam, Alexander Erofeev, Anders Sandvik, Vedrana Grcic, Frode Lars Jahnsen, Peter Gaustad, Kathy D. McCoy, Andrew J. Macpherson, Leonardo A. Meza-Zepeda, Finn-Eirik Johansen

**Affiliations:** 1 Department of Pathology and Centre for Immune Regulation, University of Oslo, Oslo, Norway; 2 Department of Pathology, Oslo University Hospital Rikshospitalet, Oslo, Norway; 3 Institute of Microbiology, University of Oslo, Oslo, Norway; 4 Maurice Müller Laboratories, Department of Clinical Research, University of Bern, Bern, Switzerland; 5 Department of Tumor Biology, the Norwegian Radium Hospital, Oslo University Hospital, Oslo, Norway; 6 Norwegian Microarray Consortium, Department of Molecular Biosciences, University of Oslo, Oslo, Norway; Charité, Campus Benjamin Franklin, Germany

## Abstract

**Background:**

Inappropriate cross talk between mammals and their gut microbiota may trigger intestinal inflammation and drive extra-intestinal immune-mediated diseases. Epithelial cells constitute the interface between gut microbiota and host tissue, and may regulate host responses to commensal enteric bacteria. Gnotobiotic animals represent a powerful approach to study bacterial-host interaction but are not readily accessible to the wide scientific community. We aimed at refining a protocol that in a robust manner would deplete the cultivable intestinal microbiota of conventionally raised mice and that would prove to have significant biologic validity.

**Methodology/Principal Findings:**

Previously published protocols for depleting mice of their intestinal microbiota by administering broad-spectrum antibiotics in drinking water were difficult to reproduce. We show that twice daily delivery of antibiotics by gavage depleted mice of their cultivable fecal microbiota and reduced the fecal bacterial DNA load by 400 fold while ensuring the animals' health. Mice subjected to the protocol for 17 days displayed enlarged ceca, reduced Peyer's patches and small spleens. Antibiotic treatment significantly reduced the expression of antimicrobial factors to a level similar to that of germ-free mice and altered the expression of 517 genes in total in the colonic epithelium. Genes involved in cell cycle were significantly altered concomitant with reduced epithelial proliferative activity *in situ* assessed by Ki-67 expression, suggesting that commensal microbiota drives cellular proliferation in colonic epithelium.

**Conclusion:**

We present a robust protocol for depleting conventionally raised mice of their cultivatable intestinal microbiota with antibiotics by gavage and show that the biological effect of this depletion phenocopies physiological characteristics of germ-free mice.

## Introduction

The human gut harbors a microbiological community of 10^14^ bacteria consisting of more than a 1000 species whose collective genome (“microbiome”) outnumbers the human genome by more than a 100 fold[1], [2]. The cross talk between the host and its intestinal microbiota is of obvious significance for the function of the intestines[3], and has also been shown to be important for immune-mediated diseases with extra-intestinal manifestations such as in allergy and asthma[4]. More recently the influence of the intestinal microbiota in experimental models for both type 1 diabetes, obesity, and multiple sclerosis has been demonstrated[5]–[7]. Microbe-associated molecular patterns (MAMPs) use an array of germ-line encoded pattern recognition receptors (PRRs) to activate signaling pathways in host cells [8], [9]. Importantly, signaling via these receptors drive host inflammatory responses to microbes[10]–[12], but can also mediate mucosal homeostasis and integrity when the microbe or microbial product is delivered via the intestinal lumen to the intact epithelium[8], [9]. Functional assessments of the intestinal microbiota's impact on the host is thus of great interest.

The intestinal epithelium, consisting of a single cell layer, composes the barrier between the host sterile environment and the bacteria-rich intestinal lumen. Having only a partially bacteria-free mucus layer as a mechanical shield[13], the intestinal epithelial cells (IECs) are in direct contact with the highly diverse microbiota[14], [15]. The enterocytes express a wide range of PRRs whose functions and engagement by MAMPs are essential for the homeostasis of the intestinal mucosa[8], [9]. The other way around, IECs are shown to modulate the microbiota through the secretion of antimicrobial peptides[16] (and reviewed in ref[15]) and by facilitating the transport of secretory IgA[17], [18]. Even though the close and mutual relationship between the intestinal microbiota and the IECs is demonstrated, very little knowledge exists about how the full gene expression repertoire in the colonic IECs is regulated by the presence of a complete conventional microbiota.

Germ-free animals bred in sterile environment may potentially be used for such comparative studies. However, establishing and running germ-free facilities is expensive and requires special expertise and infra-structure. Compared with animals living in a conventional microbiological environment, germ-free animals display an immature and underdeveloped lymphoid system[19].

A generally accessible alternative to using germ-free animals for studying host-microbe interaction *in vivo* is to deplete animals of their intestinal microbiota by using a combination of broad spectrum antibiotics administered *per os*. In a hallmark paper, Rakoff-Nahoum and colleagues delivered broad-spectrum antibiotics in the drinking water to demonstrate that intestinal MAMPs signaling via Toll-like receptors (TLRs) supported gut homeostasis[20]. However, many have found it difficult to reproduce this protocol, and researchers who have succeeded in making mice drink the antibiotics *ad libitum* have experienced increased baseline morbidity and mortality among some strains and genotypes of mice subjected to this protocol (W. Garrett, personal communication). Importantly, several published papers that report to have applied this intestinal microbiota depletion protocol describe incomplete depletion of the cultivable bacteria[21]–[23].

In order to compare the gene expression profiles of colonic IECs in the presence and absence of a complete intestinal microbiota, we determined to refine a protocol that would deplete mice of their cultivable intestinal microbiota in a predictable and reproducible manner while ensuring the health of the animals. We demonstrate that applying this refined protocol we obtain mice which resemble germ-free mice in terms of hypoplastic lymphoid tissue. Importantly, antibiotic-gavaged mice have significantly altered gene expression profiles of colonic IECs resembling those of germ-free mice and affecting a wide range of pathways.

## Results

### Delivering antibiotics by gavage ensures health of the mice

**Figure 1:**
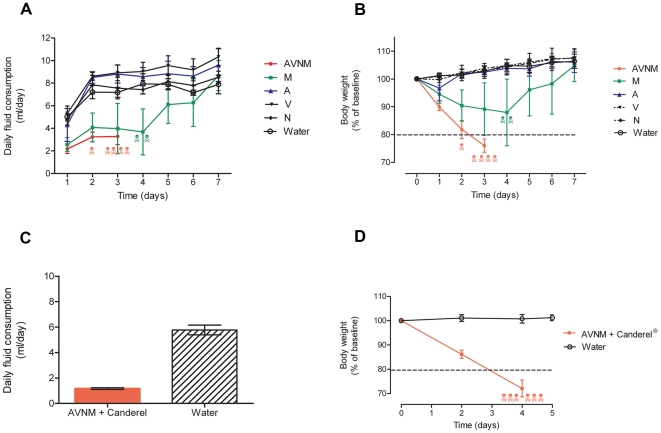
Mice refrain from drinking antibiotics *ad libitum*. Experiments attempting to administer mice ampicillin 1 g/l (A), vancomycin 500 mg/ml (V), neomycin 1 g/l (N), and metronidazol 1 g/l (M) in drinking water *ad libitum*. Water indicates mice receiving regular drinking water. Skulls represent euthanized animals. Stapled lines indicate humane endpoint of 20% loss of body weight. Treatment groups presented as mean ± SD. Deceased mice excluded from subsequent time points after death. (**A**) Daily fluid consumption estimated by daily weighting of flasks and (**B**) bodyweight presented as percent of baseline ( =  Day 0) for mice receiving the full AVNM concoction (red) in drinking water as well as for mice receiving the individual antibiotics as single solutions in drinking water, n = 5 for all groups. (**C**) Daily fluid consumption and (**D**) bodyweight presented as percent of baseline ( = Day 0) attempting to mask the foul taste of the antibiotic concoction AVNM in drinking water by adding the aspartame-based sweetener Canderel® 1.5% weight/volume, n = 6 for AVNM + Canderel® 1.5% group, n = 4 for water control group.

We attempted to deplete mice of their intestinal microbiota by providing ampicillin, vancomycin, neomycin, and metronidazol *ad libitum* in drinking water according to the previously published protocol[20]. However, BALB/c mice consistently refrained from drinking the concoction, presumably due to the foul taste of metronidazol (Figure 1A). Respecting the ethical standards of our animal facility and the humane end point of >20% loss of baseline body weight, we had to euthanize all mice receiving the full antibiotic combination (Figure 1B). Adding low caloric aspartame-based sweetener with the concoction in an attempt to mask the taste of the antibiotics also failed (Figure 1C, D). C57BL/6 mice displayed the same unwillingness as BALB/c mice to drink the antibiotic concoction provided *ad libitum* (data not shown).

**Figure 2:**
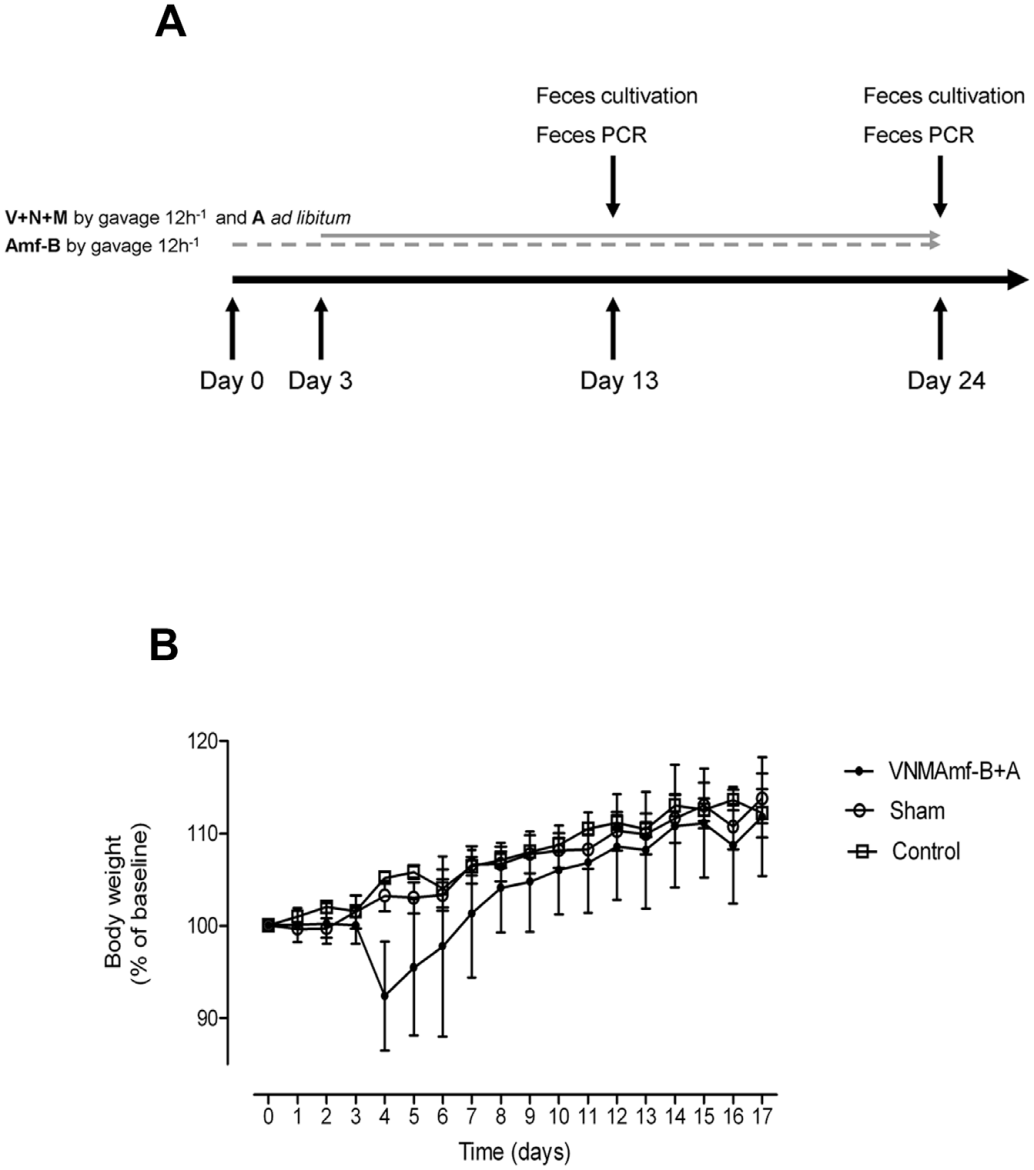
Protocol for administering antibiotics by gavage ensures mice health. (**A**) Sketch of protocol for gavage administration of vancomycin 50 mg/kg (V), neomycin 100 mg/kg (N), metronidazol 100 mg/kg (M), and amphotericin-B 1 mg/kg (Amf-B) every 12 hours with ampicillin 1 g/l *ad libitum* in drinking water (A). (**B**) Body weight, presented as percent of base line ( = Day 0), of mice successfully depleted of cultivable fecal microbiota by gavage (VNMAmf-B+A) or gavaged with water in equivalent volume and frequency (Sham). Control mice received water *ad libitum* only

To ensure a safe and stable delivery of the antibiotic concoction to every mouse subjected to the protocol we administered it by gavage every 12 hours. Due to occasional overgrowth of *Candida* spp. in pilot experiments (data not shown), we initiated our protocol with 3 days treatment of gavaging the antifungal substance amphotericin-B (1 mg/kg bodyweight (BW)) attempting to suppress fungal growth prior to starting the antibacterial treatment (Figure 2A). From day 3, ampicillin 1 mg/ml was added to drinking water and mice were gavaged every 12 hours with the antibiotic concoction consisting of vancomycin 50 mg/kg BW, neomycin 100 mg/kg BW, metronidazol 100 mg/kg BW, and amphotericin-B 1 mg/kg BW.

Gavaging mice did not inflict detectable distress or pain. Six weeks old mice given water gavage of a volume and frequency identical to the mice receiving the antibiotic concoction displayed unaltered weight gain compared with mice receiving water *ad libitum* only (Figure 2B). Some mice gavaged with antibiotic concoction experienced a transient weight loss on day 4–5 after introduction of the antibacterial therapy (Figure 2B). This transient weight loss we believe was caused by mice adjusting to ampicillin in drinking water rather than the gavaged concoction as we observed that mice receiving ampicillin in drinking water as only therapy had the same transient drop in body weight (Figure 1B). Within a week mice receiving the full antibiotic therapy had regained their weight compared with their untreated peers and appeared healthy.

### Antibiotic therapy by gavage effectively depletes cultivable microbiota and reduces fecal bacterial DNA load

**Figure 3:**
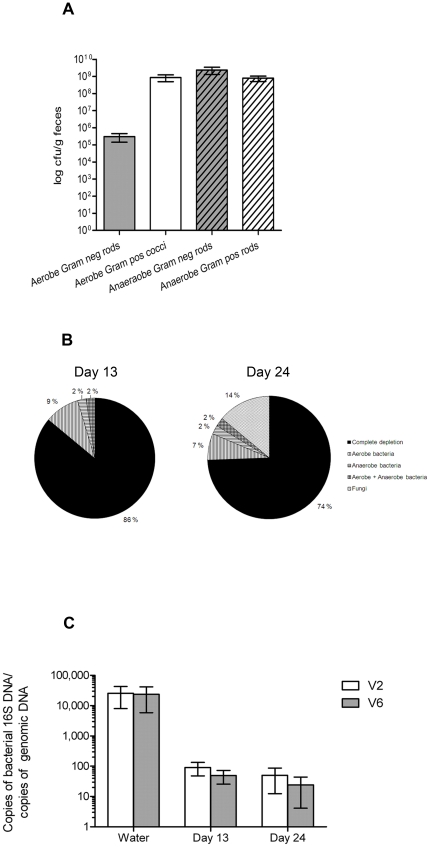
Protocol for administering antibiotics by gavage effectively reduces intestinal bacterial contents. (**A**) Enumeration of cultivable fecal bacteria (mean ± SEM) in untreated mice. (**B**) Efficacy of successful depletion (<1 cfu/mg feces) of cultivable gut microbiota and distribution of aerobe, anaerobe, and fungal overgrowth (def.: ≥1 cfu/mg feces) after 13 and 24 days of treatment with vancomycinm (V), neomycin (N), metronidazol (M), and amphotericin-B (Amf-B) every 12 hours by gavage with ampicillin *ad libitum* in drinking water (A), n = 43. (**C**) Bacterial 16S DNA load (mean ± SD) in fecal pellets. 16S DNA determined by quantitative PCR for the V2 and V6 regions and normalized to total mouse genomic DNA in the same pellets of mice treated 13 and 24 days with VNMAmf-B +A compared with water controls, n = 7 for water controls, 40 for 13 days, and 35 for 24 days.

Fecal bacteria of untreated mice were enumerated by cultivations of serial dilutions of resuspended fecal pellets on differential media (Figure 3A). Validation of successful depletion after the antibiotic gavage treatment was performed by cultivation of fecal pellets collected and handled aseptically on day 13 and 24. Due to limitations in the quantity of fresh fecal pellets that could be collected, the detection limit for this assay was set to 1 cfu/mg feces and mice with < 1cfu/mg feces were defined as successfully depleted. Only animals successfully depleted by this criterion were included in further characterization of antibiotic treated mice. At day 13, 86% of the mice subjected to the protocol displayed successful depletion of their cultivable aerobic and anaerobic fecal microbiota (Figure 3B). At day 24, the corresponding fraction was 74%. Thus, in depleted mice (<1 cfu/mg feces), we obtained a minimum of 100-fold reduction of cultivable aerobic Gram negative rods and 10^6^-fold reduction of cultivable aerobic Gram positive cocci as well as cultivable anaerobic fecal bacteria. A similar depletion efficacy was obtained in C57BL/6 mice as in BALB/c mice (data not shown).

As the majority of the intestinal microbiota is not cultivable[1] we estimated the load of bacterial DNA in feces by 16S rRNA gene quantitative polymerase chain reaction (qPCR). DNA from fecal pellets was isolated and the V2 and the V6 region of bacterial 16S rRNA genes amplified with degenerate primers targeted to conserved flanking sequences. Due to shedding of epithelial cells, feces contain host DNA. Taking advantage of this we quantified mouse DNA by qPCR and used this value to normalize the amount of bacterial 16S DNA present (Figure 3C). We found that all mice subjected to the depletion protocol had significantly reduced copy number of 16S rRNA genes in their feces: the level of bacterial DNA was similar in all samples of antibiotic treated mice and, on average, more than 400 fold less than the level in untreated mice demonstrating the effect of the microbiota depletion protocol.

### Depletion of gut microbiota produces a macroscopically germ free-like phenotype

**Figure 4:**
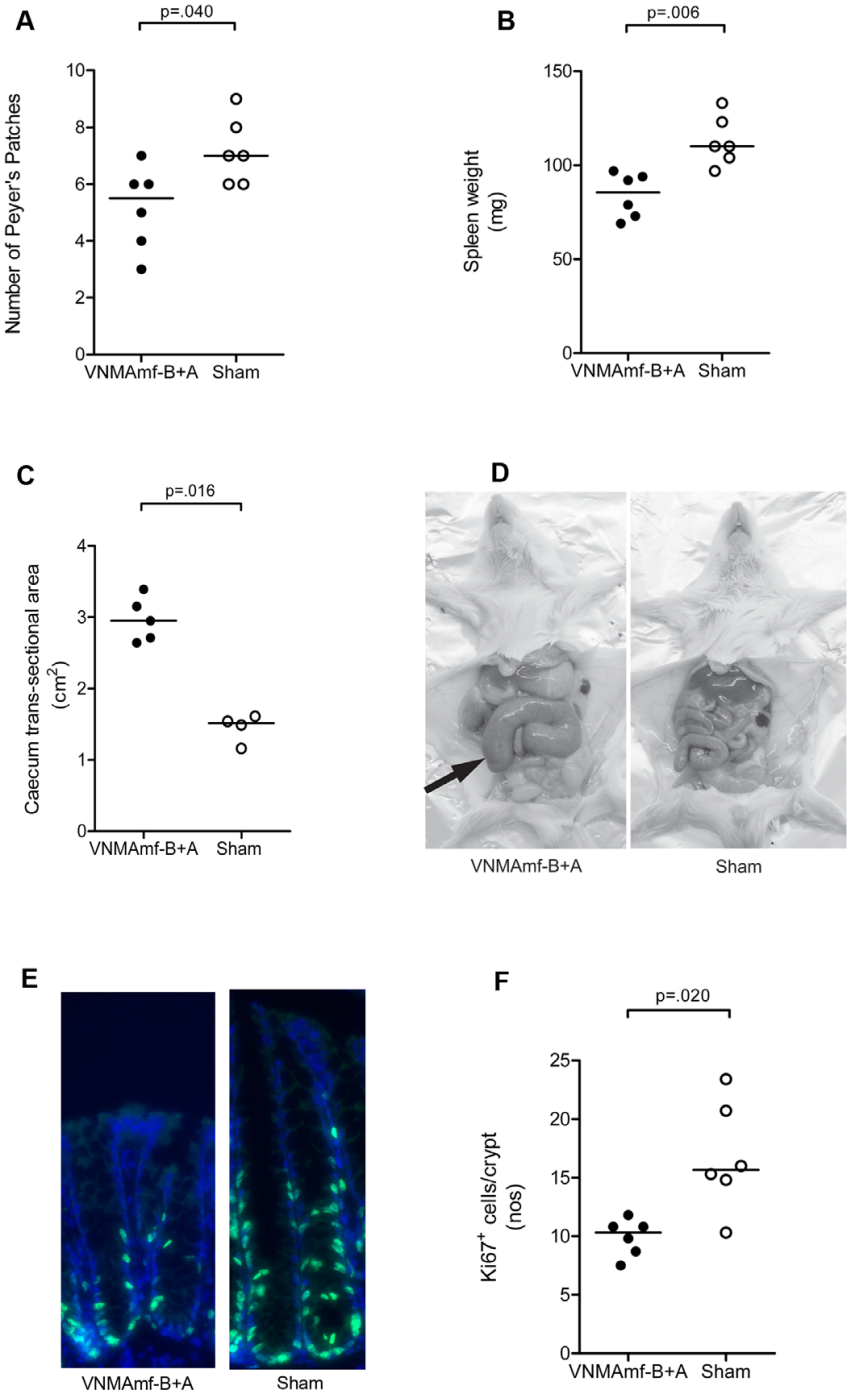
Mice subjected to antibiotic gavage treatment alters macroscopic phenotype and epithelial proliferative activity. Numbers of macroscopically visible Peyer's patches (**A**), spleen weight (**B**), and cecal longitudinal cross section area (**C**), after 17 days of treatment with vancomycin (V), neomycin (N), metronidazol (M), and amphotericin-B (Amf-B) every 12 hours by gavage with ampicillin *ad libitum* in drinking water (A) compared to mice gavaged with water only (Sham). All VNMAmf-B+A treated mice exhibited successful depletion of cultivable intestinal microbiota (<1 cfu/mg feces). (**D**) Photograph of VNMAmf-B+A and sham fed mice. Note the enlarged cecum (arrow) of the antibiotic treated mouse. (**E**) Immunofluorescent staining for Ki67 (green) and DNA (Hoechst dye; blue) of cross sections of colons from VNMAmf-B+A fed mice and sham fed mice with (**F**) enumeration of Ki67^+^ cells per crypt. In all graphs each symbol represents one animal and horizontal bars represent medians. Statistical differences calculated by Mann-Whitney two-tailed test.

Mice born and raised in a germ-free environment possess numerous characteristics distinguishing them from mice living in a conventional microbiological environment. Macroscopically, germ-free mice display hypoplastic secondary lymphoid organs, enlarged ceca, and reduced epithelial cell turnover[19]. Mice verified to be successfully depleted after treatment with the antibiotic concoction by gavage for 17 days displayed significantly fewer Peyer's patches, smaller spleens and enlarged ceca (Figure 4A–D), macroscopically phenocopying germ-free mice.

Immuno luorescent staining of sections from the colon of these same animals demonstrated that they had acquired a reduced epithelial regenerative activity in terms of fewer Ki67^+^ cells than what was observed in mice with an intact intestinal microbiota (Figure 4E, F).

### Depletion of gut microbiota alters gene expression in colonic epithelium illustrative of germ-free mice

As we observed that the refined depletion protocol affected such a basic function of IECs as the regenerative activity, we wanted to study how the intestinal microbiota affected IECs globally by performing gene expression profile analysis on isolated colonic IECs of mice successfully depleted by the refined microbiota depletion protocol compared with that of sham treated control mice. We identified a total of 517 differentially expressed genes. However, many of these genes only showed a small alteration in expression level between antibiotic treated and control animals.

**Figure 5:**
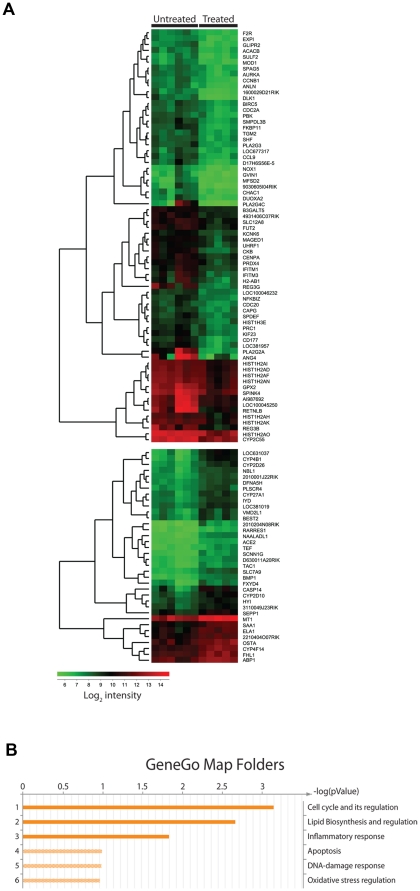
Antibiotic gavage treatment alters gene expression profile of the colonic epithelium. Gene expression profile of colonic epithelial cells isolated from mice subjected to 17 days of treatment with vancomycin, neomycin, metronidazol, and amphotericin-B every 12 hours by gavage with ampicillin *ad libitum* in drinking water (Treated) compared to mice gavaged with water only (Untreated). All Treated mice exhibited successful depletion of cultivable intestinal microbiota (<1 cfu/mg feces). (**A**) Heat-map analysis of significantly (p<0.05) differentially expressed genes with >two-fold change organized in dendrogram according to Eukledian relation of the differentially expressed genes. Colors indicate absolute expression of one gene estimated by mean values of the multiple probes detecting each gene on the chip. Each row represent one gene, each column represent one mouse. (**B**) Gene ontology map folder enrichment analysis of differentially expressed genes in MetaCore (GeneGo, St Joseph, MI).

Focusing on genes with at least a 2-fold altered expression 36 genes were higher expressed and 70 genes showed reduced expression upon antibiotic treatment (Figure 5A). Gene ontology analysis revealed that expression of genes related to cell cycle and lipid biosynthesis were the most affected of the antibiotic treatment but also inflammatory response genes were significantly altered (Figure 5B). Strikingly, 5 of the 7 genes that had more than 4-fold reduced expression in antibiotic treated mice encode known antimicrobial factors[24]–[29] (Table 1).

**Table 1:** Genes ≥ 4-fold differentially expressed in colonic epithelium of untreated conventional and antibiotic treated mice.

Gene symbol	Product	Function[Reference]	Conventional/Antibiotic treated
*Ang4*	Angiogenin, ribonuclease A family, member 4	AMF[24]	17.8
*Pla2g2a*	Phospholipase A2, group IIA	AMF[26], [34]	14.4
*Retnlb*	Resistin like beta	AMF[25], [27], [40]	8.8
*Pla2g4c*	Phospholipase A2, group IVC	AA metabolism?[41]	8.6
*Reg3g*	Regenerating islet-derived 3 gamma	AMF[25], [28]	7.6
*Reg3b*	Regenerating islet-derived 3 beta	AMF[25]	5.1
*Gsdmc2*	Gasdermin C2	Unknown[42]	4.1
*…*	…	…	…
*Casp14*	Caspase 14	Proteinase?[43]	0.23
*Cyp4b1*	Cytochrome P450, family 4, subfamily b, polypeptide 1	Metabolism of carcinogens?[44]	0.16

AMF, antimicrobial factor. AA, arachidonic acid.

**Figure 6:**
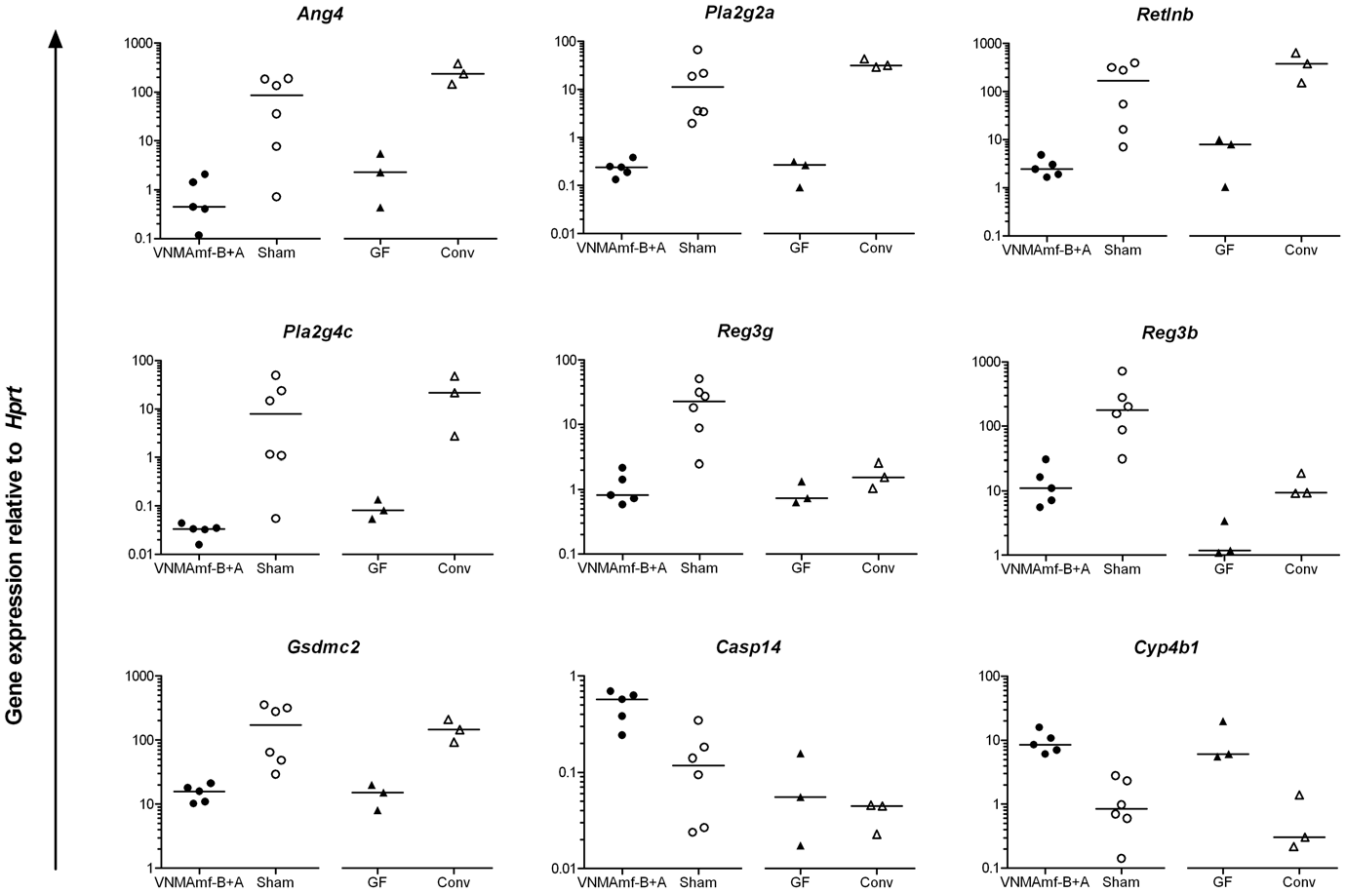
Quantitative PCR validates colonic IEC gene expression profiles of antibiotic treatment in relation to germ-free mice. Quantitative reverse transcriptase PCR of colonic intestinal epithelial cells (IEC) isolated from mice subjected to 17 days of treatment with vancomycin (V), neomycin (N), metronidazol (M), and amphotericin-B (Amf-B) every 12 hours by gavage with ampicillin (A) *ad libitum* in drinking water (VNMAmf-B+A) compared to mice gavaged with water only (Sham) and from colonic IEC of untreated germ-free mice (GF) and their conventional controls (Conv). Target gene listed on top of each panel and plotted in relation to the house-keeping gene *Hprt*. Each symbol represents one mouse, horizontal bars represent medians. Mann-Whitney analyses between VNMAmf-B+A and Sham, p<0.05 for all genes.

To validate the gene expression profile and its relevance to germ-free mice we performed quantitative reverse transcriptase (qRT)-PCR on all nine genes that showed more than 4-fold differential expression by microarray analysis (Table 1). For all nine genes tested, the qRT-PCR confirmed the differential expression between antibiotic-gavged and sham-treated mice discovered in the array (Figure 6). In all cases, the fold difference between the two groups was greater when gene expression was determined by qRT-PCR than by micro array. Moreover, differential gene expression of colonic IEC from germ-free mice versus conventional age- and gender matched controls showed similar pattern as the antibiotic-gavaged versus sham treated mice. For most of the genes the antibiotic treated and germ-free mice showed similar expression levels (Figure 6).

## Discussion

Recently, there has been an increased understanding of the importance of intestinal microbiota for human physiology, evidenced by large endeavors such as the MetaHIT project[2] and the Human Microbiome Project[30]. However, simple protocols for manipulation of intestinal microbiota in experimental animals are also needed. Thus, the seemingly simple method of depleting mice of their cultivatable microbiota by adding a concoction of antibiotics in drinking water has gained a lot of attention, but also caused a lot of frustration among immunologists. Although some labs have successfully used the *ad libitum* protocol to obtain valuable data, it appears that it is not applicable to all mouse strains or genotypes and that it is affected by the general conditions in the vivarium. Here we have presented and validated an alternative method which deliver the antibiotics in a safe and predictable manner without inflicting any morbidity on the animals. Furthermore, we demonstrated that mice subjected to this depletion protocol acquire a germ free-like phenotype.

Our protocol for depletion of intestinal microbiota is based on the same composition of antibacterial agents as others have applied for *ad libitum* administration in drinking water. Ampicillin, vancomycin, neomycin and metronidazol offer in combination bactericidal activity against the full spectrum of bacteria and, notably, dual activity against both Gram positive (ampicillin and vancomycin) and Gram negative (ampicillin and neomycin) aerobic and facultative strains, which is potentially important for preventing antibiotic resistance [31]. We therefore used this combination of antibiotics and overcame the delivery problems by administering the antibiotics by gavage twice daily which the mice tolerated well.

In mice treated with the gavage protocol for 17 days we observed a reduction in the bacterial DNA load that was substantial though numerically not as great as the reduction observed in cultivation assays. This may reflect that the 16S DNA qPCR detect DNA from dead as well as viable bacteria. Also, a recent report demonstrated that bacterial DNA in the animal chow contributes to the 16S DNA load in fecal pellets[32]. However, the fact that the few fecal samples in our assays that contained cultivable bacteria did not distinguish themselves in terms of higher 16S DNA load than the successfully depleted mice (not shown) indicates that the qPCR assay is a more crude analysis.

Fecal pellets were used for evaluating the efficacy of the microbiota depletion protocol. Microbes that preferentially adhere to the mucosa of the mice are also affected by antibiotics[32] but would not be thoroughly assessed by analysis of fecal pellets. Even though the fecal pellets were devoid of cultivable bacteria and had greatly reduced amount of bacterial DNA we acknowledge that the intestines of the mice likely harbor residual bacteria and should not be regarded as sterile, as in true germ-free mice. However, collecting fecal pellets is non-invasive and allows for longitudinal studies. Contrary to this, collecting intestinal tissue from mice to confirm depletion of adherent bacteria would not be compatible with having the same mice entering functional experiments. Applying the gavage antibiotic depletion protocol had a profound effect on the phenotype of the mice as they developed several characteristics typical of germ-free mice. The reduced spleen, number of Peyer's patches, the enlarged ceca, and reduced epithelial proliferative activity in our treated mice is also observed in germ-free mice[19]. This suggests that these features are typical of under-stimulation by commensals and that they are reversible effects rather than a consequence of growing up germ free. Furthermore, both mucosal and systemic secondary lymphoid organs were affected by commensal depletion.

We assessed the impact of the intestinal microbiota on the colonic IEC's gene expression profile, which revealed that more than 500 genes were differentially expressed as a consequence of the antibiotic by gavage treatment. A number of genes related to cell cycle were differentially regulated between untreated and antibiotic treated mice, suggesting that the commensal microbiota regulate epithelial proliferation in the colon. Furthermore, pathways regulating lipid biosynthesis, specifically production of arachidonic acid, appeared to be affected by the commensal microbiota. This was due to reduced expression of several small secreted phospholipases from the phospholipase A2 family after antibiotic treatment. Recent reports suggest that these secreted phosphoslipases are not primarily involved in arachidonic acid production. Although they have enzymatic activity, they show no substrate specificity for arachidonic acid release[26], [33]. Furthermore, secreted phospholipases have been shown to have antimicrobial activity[26], [34].

In colonic IEC from germ free mice and matched conventional mice we determined the expression level of nine genes strongly affected by our antibiotics by gavage treatment. In eight out of nine cases, differences between antibiotic and sham treated mice were nearly identical to differences between germ-free and conventional mice strongly suggesting that the altered gene expression profile is a consequence of the highly reduced microbial stimulation of the colonic IECs. For these eight genes we rule out that altered gene expression due to antibiotic treatment was a consequence of a direct pharmacologic effect of the antibiotics. These comparisons support the validity of the antibiotic treatment as an analogue to germ-free status in mice.

The greatest fold change was seen in genes coding for antimicrobial factors, some of which (*Ang4* [24], *Retnlb* [25] *Reg3g* [28] and *Reg3b* [25]) have been demonstrated to be induced by microbial stimuli through mono-associating or conventionalizing germ-free mice or by comparing germ-free and conventionally bred mice. *Retnlb, Reg3g,* and *Reg3b* have also previously been shown to be down-regulated after antibiotic treatment[25], [35]. These studies have been performed on samples from the small intestines and we now complement these by demonstrating the identical effect in the colon. We believe we are also the first to demonstrate that in the colon high expression of the antimicrobial factor *Pla2g2a* is dependent on the presence of the intestinal microbiota, which is incongruent to previous observations in the small intestines[24]. The reduced expression of antimicrobial factors in IEC from mice depleted of commensal microbiota (to levels similar to that of germ free mice) demonstrate that continuous presence of microbial stimuli is required to maintain normal expression level of these genes.

To our knowledge only one study has performed gene expression profile comparison on colonic IECs from germ-free and conventionally bread mice. In the infancy of array-based gene expression assays Fukushima *et al*. compared mRNA expression profiles of colonic IEC from germ-free mice with mice with conventional non-pathogenic microbiota and confirmed two genes downregulated and five genes upregulated in germ-free mice[36]. Of these seven genes four were similarly differentially expressed in our microbiota depleted mice. This comparison lends further support to our hypothesis that the differentially expressed genes in our antibiotic treated mice are caused by greatly reduced microbial stimuli.

Two recent publications reported to having gavaged mice with ampicillin, vancomycin, neomycin and metronidazol[7], [32]. In line with our experiments, Hill *et al*. demonstrated the pitfall of the previously reported antibiotics *ad libitum* protocol and showed that gavaging mice with antibiotics once daily gave a significant reduction in bacterial 16S DNA load and altered gut phenotype[32]. Ochoa-Reparaz *et al.* demonstrated that mice gavaged with the antibiotics tolerated that treatment better than the *ad libitum* approach[7]. Our protocol applied a gavage frequency of twice daily and we used higher antibiotic concentrations than Hill *et al.* and Ochoa-Reparaz *et al.* Thus, we were able to reduce the fecal 16S DNA load by 400 fold while Hill *et al.* reported a 10-fold reduction. We have furthermore validated the gavage protocol demonstrating acquisition of a “germ free-like” phenotype of the subjected mice. We believe that our assessments of the macroscopic appearance of lymphoid organs and colonic IEC gene expression profile provide additional knowledge on how the intestinal microbiota affects a mammalian organism.

In conclusion, we present a generally accessible protocol for depleting mice of their cultivable intestinal microbiota by administering an antibiotic concoction by gavage every 12 hours. Our protocol showed increased feasibility compared with previous protocols, provided a predictable delivery of the antibiotics and at the same time ensuring the health of the animals. We have verified the depletion efficacy of our protocol both in terms of cultivable microbes and in terms of bacterial DNA load in feces. Finally, our protocol proved to produce a germ free-like phenotype of the animals subjected to the protocol, suggesting it to be a valid and accessible way to perform experiments on the host-bacteria cross talk in mice.

## Materials and Methods

### Ethics statement

All use of laboratory animals was approved by the National Animal Research Authority (Forsøksdyrutvalget) (approval IDs: 48/05, 1468, and 1734) and conducted in accordance with the Norwegian Animal Welfare Act and the Norwegian Regulation on Animal Experimentation. Humane endpoint was set to loss of >20% body weight compared with the starting weight in each experiment.

### Mice

BALB/c mice were bred and kept in a conventional laboratory animal facility at Centre for Comparative Medicine, University of Oslo with temperatures maintained at 21^o^C and with 55% relative humidity, 12 hour light and darkness cycles with 1 hour of dusk and dawn. The mice received regular chow No. 3 (801080, Special Diets Services, Witham, England) and water purified by reverse osmosis and ionic exchange. Males 6–10 weeks of age were used in experiments, age and weight matched for each individual experiment to have an age span of maximum 2 weeks and a weight range of maximum ±20% of medians.

For reference controls in quantitative gene expression analyses 6 month old female BALB/c were obtained from the Clean Mouse Facility (CMF), Department of Clinical Research, University of Bern. Germ-free mice were bred and maintained in flexible film isolators and germ-free status was routinely confirmed by aerobic and anaerobic culture as well as DNA (sytox green; Invitrogen) and gram staining (Harleco) of cecal contents to detect unculturable contamination. Germ-free BALB/c mice were sacrificed under sterile conditions and tissue collection was performed at the University of Bern. Age- and gender matched conventionally kept controls were from Centre for Comparative Medicine, University of Oslo.

### Antibiotic treatment protocol

Antibiotic treatment started with three days of amphotericin-B (Bristol Meyers Squibb, New York City, NY) 0.1 mg/ml, administered by gavage 12h^−1^ (Figure 2A). From day three, water flasks were supplemented with ampicillin (Bristol Meyers Squibb, New York City, NY) 1 g/l and antibiotic concoction consisting of vancomycin (Abbot, Abbot Park, IL) 5 mg/ml, neomycin (Invitrogen, Carlsbad, CA) 10 mg/ml, metronidazol (Actavis, Hafnarfjordur, Iceland) 10 mg/ml, and amphotericin-B (Bristol Meyers Squibb) 0.1 mg/ml was administered by antibiotic gavage 12h^−1^. Gavage volume of 10 ml/kg body weight was delivered with a stainless steel tube without prior sedation of the mice. Fresh antibiotic concoction was mixed every day and ampicillin and water was renewed every 7th day.

### Bacterial cultivation of feces

Day 13 and day 24 of the antibiotic treatment mice were fixed to defecate directly into a pre-weighted 2 ml capped microtube (Sarstedt, Nümbrect, Germany) prefilled with 1 ml sterile ice-cold phosphate-buffered saline (PBS). Tubes with fecal pellets were kept on ice, weighed and the weight of the pellets calculated (median 46 mg, range 17–120). Fecal pellets were resuspended in the 1 ml PBS by vortexing and by bashing with a sterile bacteriological loop. The fecal suspension was then plated on blood agar, anaerobic blood agar (hemin – vitamin K agar), and yeast agar (Sabouraud agar) in doubles with 100 µl suspension on each plate. Blood agar and Sabouraud agar plates were incubated aerobically at 37°C with 5% CO_2_ for 72 hours, while anaerobic blood agar plates were incubated at 37°C in anaerobic conditions for 96 hours. At the end of incubation the numbers of colonies on the plates were counted and the number of bacteria per mg of feces calculated. Evaluation of cultivated agar plates was performed by an experienced bacteriologist (P.G.) The detection limit of the assay was defined as 1 cfu/mg feces. Only mice successfully depleted (<1 cfu/mg feces) were included in phenotypic and gene expression analyses.

As a positive control for the depletion verification assay, and to enumerate cultivable microbes with the fecal collection procedure, fecal pellets from untreated mice were collected with the above described procedure. Serial dilutions made in sterile PBS and suitable dilutions were plated on selective media for intestinal Gram negative rods, enterococci, anaerobic Gram negative rods (*Bacteroides* spp), *Clostridium* spp, *Lactobacillus* spp and *Bifidobacterium* spp. The aerobic agar plates were incubated in 37°C with 5% CO_2_ for 48 hours while anaerobic agar plates were incubated in 37°C for 48 hours. After incubation the numbers of colonies on the plates were counted and the number of bacteria per mg of feces was calculated.

### 16S rRNA gene quantification

In the same procedure as fecal pellets were collected for cultivation, the mice were let to defecate directly into a dry 1.5 ml capped microtube (Sarstedt, Nümbrect, Germany) which was snap frozen in liquid nitrogen and stored at −70°C until use.

DNA was isolated from bacterial fecal pellets with QIAamp DNA Stool Mini Kit (Qiagen, Hilden, Germany) and quantified by spectrophotometry at 260 nm. Degenerate primers for V2 and V6 region of bacterial 16S genes were as previously described[37], [38] and primers for mouse genomic DNA was designed with Primer3 (frodo.wi.mit.edu) (Table 2). All PCR reactions were carried out with 50 ng of DNA, EvaGreen® (Biotium, Hayward, California) and Taq2000 in a Stratagene MX3000P with a 15 min activation step (95°C); then 40 cycles of 30 s denaturation (95°C), 30 s annealing (60°C for 16S-V2 and mpIgR-genomic, 55°C for 16S-V6), and 30 s extension (72°C). The efficiency of each PCR was determined by dilution series of template. The number of 16S DNA copies was related to the number of mouse genomic DNA copies for each sample. Samples with a threshold crossing point >32 cycles for the genomic PCR were deemed poorly amplifiable and excluded from analysis.

**Table 2:** PCR primers used for amplification of bacterial 16S, mouse genomic DNA, and colonic IEC target genes.

Target	Primer name	Sequence
16S V2 region	16S-V2-101F[^37^]	AGYGGCGIACGGGTGAGTAA
	16S-V2-361R	CYIACTGCTGCCTCCCGTAG
16S V6 region	16S-V6-784F[^38^]	AGGATTAGATACCCTGGTA
	16S-V6-1061R	CRRCACGAGCTGACGAC
Mouse pIgR genomic region	mpIgRgenomic.for	TTTGCTCCTGGGCCTCCAAGTT
	mpIgRgenomic.rev	AGCCCGTGACTGCCACAAATCA
Angiogenin, ribonuclease A family, member 4	Ang4.for	TCTCCAGGAGCACACAGCTA
	Ang4.rev	AAGGACATGGGCTCATTGTC
Phospholipase A2, group IIA	Pla2g2a.for	CTGTTGCTACAAGAGCCTGG
	Pla2g2a.rev	TTTTCTTGTTCCGGGCGAAA
Resistin like beta	Retnlb.for	AGGATCAAGGAAGCTCTCAGTC
	Retnlb.rev	ATTTCCATTCCGGATATCCCA
Phospholipase A2, group IVC	Pla2g4c.for	AAGGCTCTCAGACTGTGGAG
	Pla2g4c.rev	GCCCACAGTACCCTGAAAAC
Regenerating islet-derived 3 gamma	Reg3g.for	ACATCAACTGGGAGACGAATC
	Reg3g.rev	TTTGGGATCTTGCTTGTGGCTA
Regenerating islet-derived 3 beta	Reg3b.for	CCTTAGACCGTGCTTTCTGTG
	Reg3b.rev	GTCCATGATGCTCTTCAAGACA
Gasdermin C2	Gsdmc2.for	GGACCTGGAGGCTAACTTGA
	Gsdmc2.rev	CCTTTCCATCCGGCAAAACT
Caspase 14	Casp14.for	ATCTCAGGAGAAGCTTGGGG
	Casp14.rev	TCTGGCTTTCAGCACCTTTG
Cytochrome P450, family 4, subfamily b, polypeptide 1	Cyp4b1.for	TGATCCTGATGGTAACCGTCC
	Cyp4b1.rev	CATAGGGGAACTGTTCGGTC
Hypoxanthine guanine phosphoribosyl transferase	HPRT.for	TGATCAGTCAACGGGGGACA
	HPRT.rev	TTCGAGAGGTCCTTTTCACCA
Beta 2 microglobulin	B2m.for	TGACCGGCCTGTATGCTATC
	B2m.rev	GCAGTTCAGTATGTTCGGCT

Target region, name and sequence of primers used in this study. The matching forward and reverse primers targeted to mouse cDNA lie in separate exons of each gene.

### Organ evaluation

After 17 days of antibiotic treatment mice were anaesthetized with 150 µl Hypnorm® (fentanyl citrate 0.315 mg/ml and fluanison 10 mg/ml, VetaPharma Ltd, Leeds, UK) and midazolam (5 mg/ml, B. Braun Melsungen AG, Melsungen, Germany) subcutaneously and sacrificed by cardiac puncture. Abdominal organs were swiftly dissected and evaluated by a technician blinded to the treatment of the individual animal. Macroscopically visible Peyer's patches along the entire length of the small intestines from the ventricle to the ileocecal junction were counted. Cecum was gently put on its side along a ruler, digitally photographed and with the software analySISpro (Olympus Europa, Hamburg, Germany) the longitudinal cross section area of the cecum was calculated. The intact spleen was excised, abdominal fat gently removed, and the spleen was put in 10% formalin for 24 h, and then removed from the formalin, excess liquid wiped off with a filter paper, and the spleen was weighted.

### Immunohistochemistry

Colons of sacrificed mice were swiftly dissected and flushed with 2× 10 ml of ice cold PBS. A 1.5 cm piece of the middle colon was cut off and instantly fixed in 10% formalin for 24 h at 4°C before being automatically processed (TP 1050, Leica Microsystems, Wetzlar, Germany) and embedded in paraffin. Sections were cut at 4 µm thickness, deparaffinized and incubated 20 minutes in preheated 0.05% citraconic anhydride pH 7.4 at 98°C. After cooling to room temperature the sections were incubated 18 h with rabbit anti-Ki67 (Abcam, Cambridge, UK) or isotype control (rabbit anti-hemocyanin, Sigma, St. Louis, MR) at 4°C. After washing with PBS (pH 7.4) sections were incubated 90 minutes with Alexa Fluor 488 conjugated goat anti-rabbit IgG (Molecular Probes, Eugene, OR) at room temperature. After another washing in PBS sections were stained with Hoechst dye before being rinsed in water and mounted with PVA under cover slips.

Slides were evaluated by one examiner (D.H.R) a blinded fashion with the help of microscope (Eclipse E800, Nikon, Tokyo, Japan) fitted with a digital camera and imaging software (AnalySISpro 3.2, Olympus Soft Imaging System GmbH, Münster, Germany). Enumeration of Ki67^+^ cells were performed by calculating the mean number of positive cells in 5–8 crypts in each transverse section ensuring crypts were cut to display the full crypt heights.

### Isolation of colonic IECs and microarray

After 17 days of antibiotic treatment mice were anaesthetized with 150 µl Hypnorm® (fentanyl citrate 0.315 mg/ml and fluanison 10 mg/ml, VetaPharma Ltd) and midazolam (5 mg/ml, B. Braun Meslungen AG) subcutaneously and bled to death by cardiac puncture. Colon was swiftly excised and flushed with 2× 10 ml ice cold PBS (w/o Mg^2+^ and Ca^2+^) and kept moist. Mesenteric and adipose tissue was removed from the colon which was subsequently opened longitudinally, then cut transversally in 5 cm long pieces and incubated 25 min in 25 ml 20 mM EDTA (Sigma-Aldrich, St. Louis, MO) at room temperature on a shaker before being vigorously hand shaken for 5 minutes. The colonic IECs were harvested in a fresh tube, washed twice in ice cold PBS, resuspended in TRI Reagent® (Ambion Applied Biosystems, Foster City, CA), and stored at −70°C until RNA isolation according to manufacturer's instructions.

Gene expression profiling was performed at the Microarray Core Facility at The Norwegian Radium Hospital (Oslo, Norway). Total RNA corresponding to samples of 5 microbiota depleted mice and 6 control mice were labeled and hybridized to Illumina's MouseWG-6 v2.0 Expression BeadChips (Illumina, Inc., San Diego, CA) according to manufacturer's protocol. Normalization and statistical analysis of gene expression was performed in R (www.r-project.org) using Bioconductor packages and differentially expressed genes were identified using moderated t-statistics with an adjusted p-value <0.05. Differentially expressed genes were further analyzed in MetaCore (GeneGo, St Joseph, MI) to identify functional enrichment. Process and pathways were selected based on a p-value < 0.05. The complete gene expression dataset can be viewed in the Gene Expression Omnibus (GEO) repository accession number GSE22648 (www.ncbi.nlm.nih.gov/geo/query/acc.cgi?acc=GSE22648). Data submitted complied with MIAME standards.

### Quantitative RT-PCR

Epithelial cells and mRNA were isolated as described above. cDNA was synthesized with Superscript^TM^ III Reverse transcriptase (Invitrogen) and 20 pmol/µl oligo(dT) according to manufacturer's protocol. The PCR primers were selected with the “Pick PCR primer” tool available from NCBI (http://www.ncbi.nlm.nih.gov) making sure each primer pair was separated by an intron on genomic DNA. The optimal Mg^++^ concentration was determined empirically for each primer pair and PCR performed with EvaGreen® (Biotium, Hayward, California) and Taq2000 in a Stratagene MX3000P with a 15 min activation step (95°C); then 35 cycles of 30 s denaturation (95°C), 30 s annealing (60°), and 30 s extension (72°C). The median value of triplets were used to calculate relative expression of each gene according to the ΔΔCt method[39]. Normalization to the housekeeping genes *hprt* or β2-microglobulin gave similar results.

### Statistics

Where appropriate, statistical differences were calculated by Mann-Whitney two- tailed test using the software Prism5 (GraphPad Softvare Inc., La Jolla, CA).
